# Delay in loop ileostomy reversal surgery does not impact upon post-operative clinical outcomes. Complications are associated with an increased loss of microflora in the defunctioned intestine

**DOI:** 10.1080/19490976.2023.2199659

**Published:** 2023-04-13

**Authors:** Emma L. Beamish, Judith Johnson, Barbara Shih, Rebecca Killick, Frank Dondelinger, Ciarra McGoran, Caitlan Brewster-Craig, Albert Davies, Arnab Bhowmick, Rachael J. Rigby

**Affiliations:** a Division of Biomedical and Life Sciences, Faculty of Health and Medicine, Lancaster University, Lancaster, UK; b Royal Preston Hospital, Lancashire Teaching Hospitals NHS Foundation Trust, Sharoe Green Lane, Fulwood, Preston, UK; c Department of Mathematics & Statistics, Faculty of Science and Technology, Lancaster University, Lancaster, UK; d Centre for Health Informatics, Computation and Statistics, Faculty of Health and Medicine, Lancaster University, Lancaster, UK; e Furness General Hospital, University Hospitals of Morecambe Bay Hospitals NHS Foundation Trust, Dalton Lane Barrow-In-Furness, Cumbria

**Keywords:** Surgery, Post-operative, Ileus, Microbiota, dysbiosis, stoma, complications, ileostomy

## Abstract

Loop ileostomy is a common surgical procedure to allow downstream tissue healing, with the aim of re-joining the bowel approximately 12 months later. The reversal procedure is associated with a substantial morbidity up to 40%. Our previous research demonstrated that defunctioned ileum becomes atrophied, with extensive microbial dysbiosis. This study sought to investigate the potential influence of delaying ileostomy reversal surgery upon both clinical and pathological outcomes. Post-operative clinical data was recorded, including routine blood test results, duration of hospital stay, length of time with stoma and incidence of post-operative complications. We measured ileal fibrosis and atrophy and assessed whether these, or dysbiosis, were impacted by the length of time a stoma was in place, or were linked to clinical outcomes. Associations between clinical data were investigated using scatterplot matrix analysis and t-tests. We found no differences in time between ileostomy formation and reversal in patients experiencing complications vs. individuals with no complications. Furthermore, there were no correlations between days with stoma and pathological measures, such as atrophy or fibrosis, and no ongoing increases in collagen production at the time of reversal surgery. This data suggests that the length of time a stoma is in place does not impact on the likelihood of complications. The incidence of complications is associated with increased loss of microbiota in the defunctioned ileum, but importantly, the decrease in bacteria is not linked to time with stoma. Microbiota diversity in the functional and defunctioned limb correlated within an individual, and was not significantly different between those who experienced complications following surgery vs. those that didn’t. Microbiota diversity was also not significantly impacted through delay (>365 days) in stoma reversal. We propose that methods to restore intestinal microbiota numbers, and not necessarily their composition, prior to reversal should be explored to improve the clinical outcomes of ileostomy reversal surgery.

## Introduction

Loop ileostomy is a surgical procedure used to protect downstream intestinal anastomoses, commonly formed upon removal of a tumor from the colon or downstream Crohn’s disease, and is usually reversed upon anastomotic healing or remission. Although most stoma formations are reversible, some patients due to complications and various morbidities are unable to undergo, or have ongoing complications following, reversal surgery^1,2^. It is well known that the initial procedure renders the distal ileum defunctioned, atrophied, and fibrotic^3,4^. More recent research, conducted by our group, has suggested that in spite of the atrophy, there is no chronic inflammation in the defunctioned tissue based upon histological examination^5^, but loss of function induces dysbiosis, with reductions in both bacterial load and diversity^5^.

Prior to the current study, a Public and Patient Involvement event, funded by the Research Design Service North West (NIHR), provided us with a set of queries that are most important to the patients that undergo loop ileostomy surgery. The most pertinent question, according to patients, was ‘Will delaying reversal surgery increase the risk of developing complications?’ This question has become more pressing over recent years, as the SARS-CoV-2 pandemic has resulted in a considerable backlog of elective surgeries.

The reversal procedure is associated with a substantial morbidity up to 40%^6,7^. Some of the most common side effects of reversal surgery include reduction in absorption leading to symptoms, such as erratic bowel function, characterized by diarrhea and/or incontinence and bowel distension, and surgical wound infection. Around 20% of patients experience more serious complications^6^, including lack of normal muscle contractions of the intestines (ileus), inflammation (ileitis), or anastomotic leakage. Currently, we do not know what pre-operative factors are associated with an increased risk of post-operative complications, but one study suggested that surgical closure technique and interval time from ileostomy creation to closure might be relevant^8^.

Fibrosis is a physiological process triggered by the onset of inflammation that may lead either to tissue repair or pathological fibrosis depending on the balance between production of collagen and enzymatic degradation. Myofibroblasts, fibroblasts, and smooth muscle cells are involved in fibrosis. In the normal intestine, subepithelial myofibroblasts and fibroblasts in the submucosa and intermuscular connective tissue are the primary sites of collagen synthesis and deposition^9^. Collagen Type I alpha I is the most abundant collagen in the human body^10^ and is upregulated during intestinal fibrosis following ileo-cecal resection^11^.

Intestinal epithelial cells (IECs), such as those lining the crypts and villi, elicit the most immediate response to nutrient deprivation through suppression of cell growth and proliferation, as observed by Beamish et al.^5^. During prolonged periods of cellular stress, degradation, and recycling of IEC and myocytes may be required to preserve the replenishment of the tissue, albeit at a reduced rate. It is likely that nutrient deprivation activates autophagy in the affected cells and recycled amino acids derived from the degraded proteins and organelles to obtain energy. In the short term, this mechanism allows cells to remain viable. However, to maintain tissue mass the rate of breakdown must equal the rate of synthesis. Increases in autophagy have been observed in defunctioned intestine^4^ favoring atrophy of the intestinal epithelium. Prolonged lysosomal degradation of protein comprising smooth muscle within the intestine will almost certainly have detrimental impacts to the function of the muscle, accounting for the well-documented loss of contractility and strength leading to ileus^4^.

Despite the likelihood of the functional consequences of atrophy and fibrosis contributing to many of the complications observed following reversal surgery, we currently do not know whether a higher degree of atrophy and fibrosis is associated with an increased risk of complications. There is a plethora of evidence to demonstrate the role of the microbiota in maintaining intestinal function, and health^12,13^ and there is also some evidence to suggest that the microbiome may influence anastomotic leak following surgery^14^. Unsurprisingly, diversion of the fecal stream to a stoma results in significant (average 52%), but not total, loss of the resident microflora^5^. We therefore examined whether patients with increased atrophy, fibrosis, more pronounced microflora loss or dysbiosis within the defunctioned intestine would be more likely to experience complications of reversal surgery.

## Research aims

- To consider the consequences of tissue changes, such as fibrosis and atrophy, following loop ileostomy-mediated fecal stream diversion on post-operative clinical outcomes.

- To investigate correlations between factors that may influence the incidence of complications following ileostomy reversal surgery, particularly regarding changes in intestinal physiology and microbiota.

- To assess whether the length of time the temporary stoma is in place impacts on the degree of physiological changes in the intestine, or increases the risk of complications.

## Results

### Defunctioning of the intestine is associated with fibrosis and autophagy

**Figure 1.**
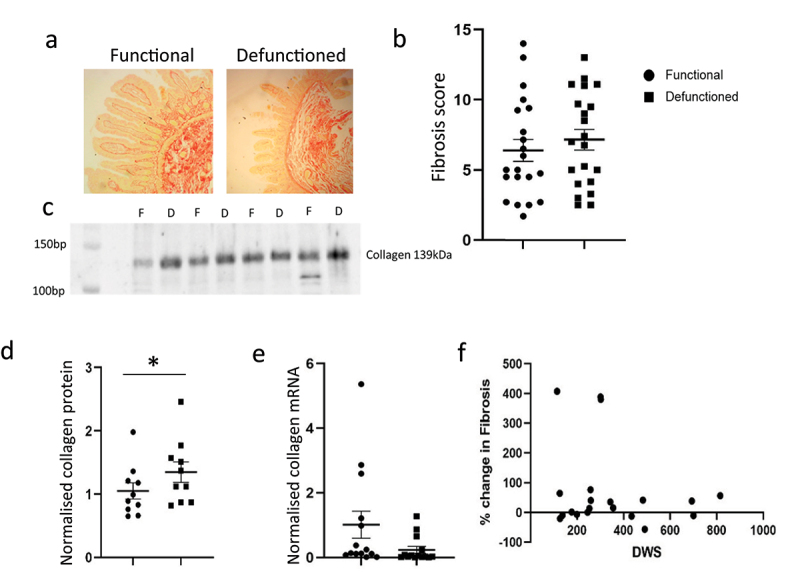
Fibrosis in the functional vs. defunctioned intestine. (a) Representative Sirius red stained sections. (b) Fibrosis scoring in functional (●) vs. defunctioned (■) ileum, *n* = 21 paired samples, line represents Mean ± SEM. (c) Representative Western blot of Collagen Type 1 in functional (F) vs. defunctioned (D) ileum. (d) Mean ± SEM Type I collagen protein normalized to total protein in functional (●) vs. defunctioned (■) ileum (*n* = 9 paired samples, *p = 0.03). (e) Mean ± SEM *COL1A1* mRNA expression normalized to rplp0 in functional vs. defunctioned ileum (*n* = 13 paired samples, *p* = 0.055). F: Days with stoma (DWS) plotted against % change in fibrosis (*n* = 21, n.S.).

**Figure 2.**
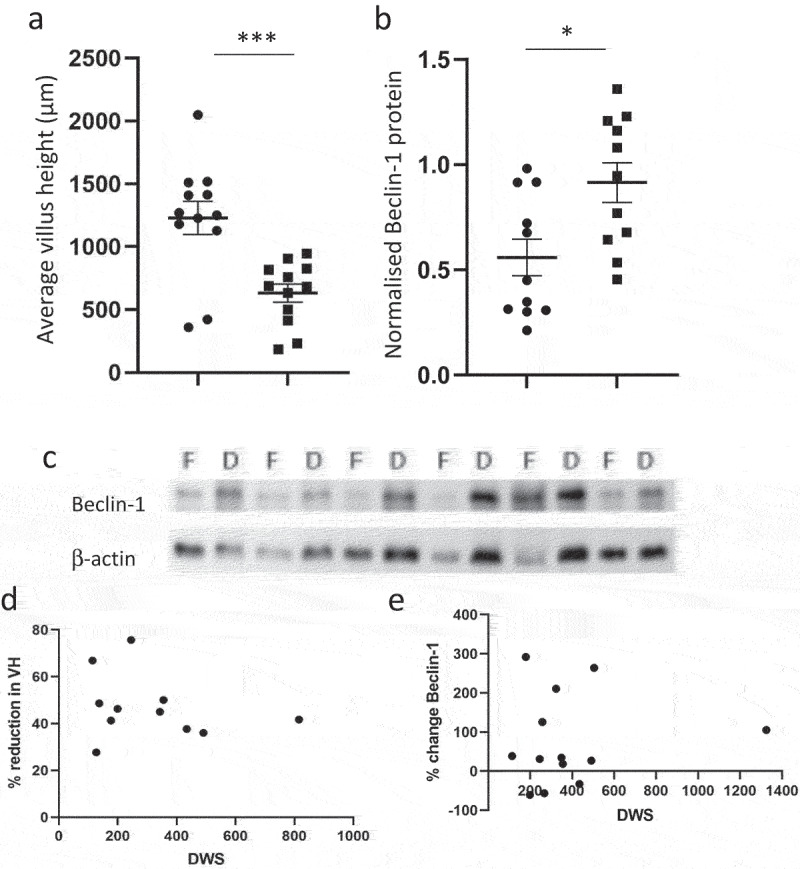
Atrophy in the functional vs. defunctioned intestine. (a) Average villus height (VH) in functional (●) and defunctioned (■) ileum (*n* = 12 paired samples, *p* = 0.0001). (b) Mean ± SEM Beclin-1 protein expression normalized to β-actin in functional (●) vs. defunctioned (■) ileum (*n* = 11 paired samples, *p = 0.003). (c) Representative Western blots of Beclin-1 and b-actin in functional (F) and defunctioned (D) ileum. (d) Days with stoma (DWS) plotted against % change villus height (VH, *n* = 11, n.S.) and E: % change Beclin-1 (*n* = 13, n.S.).

We assessed two independent measures of fibrosis through scoring of Sirius Red stained sections and quantification of collagen type I protein in functional and defunctioned tissues within individuals (Figures 1). A modest increase in average overall ‘score’ from 6.4 to 7.2 equated to an indication of no difference in fibrosis between functioning and defunctioned ileum across the cohort (Figure 1b). However, certain individuals did display marked increases in fibrosis at the time of surgery reversal and an average 22% increase in collagen type 1 expression (Figure 1d) was observed between functioning and defunctioned bowel across the cohort (*p* = 0.03). However, assessment of mRNA expression indicated that there was no ongoing collagen production at the time of reversal surgery (Figure 1e) and that levels of fibrosis did not increase with time the stoma was in place. Atrophy in defunctioned bowel was also confirmed via two measures, a reduction in villus height, average 47% (*n* = 11, *p* = 0.0001) (Figure 2a) and a concomitant 77% (*n* = 13, *p* = 0.003) increase in Beclin-1 protein, as a marker of autophagy (Figure 2b).

### Post-operative clinical data

**Figure 3.**
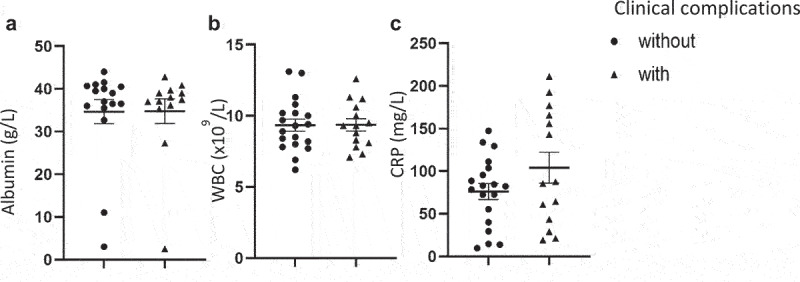
Serum albumin, White Blood cell count (WBC) and C-reactive protein (CRP) in *n* = 35 patients without (●) or with (▲) post-operative complications. Lines represent Mean ± SEM.

A combined total morbidity of 40% was observed post-operatively (Table 1), in line with published data^6^. The most common complications were surgical wounds (20%), post-operative ileus (11%), and erratic bowel function (6%). As expected following any invasive surgery, a substantial elevation in post-operative CRP levels above the normal range, with an average of 87.9 mg/L, and a concomitant reduction in serum albumin (average 38.1 g/L) was seen across the patient cohort (Table 1). None of the serum markers were associated with an increased likelihood of developing complications following stoma reversal surgery (Figure 3).

**Table 1.** Incidence of post-operative complications following ileostomy reversal surgery. #, number of patients affected; %, percent of patients affected across the cohort. N.B. Patient with anastomotic leak also counted in the Ileus category.

Post-operative Complications	#	%
Ileus	4	11.4
Anastomotic Leak (with ileus)	1	2.9
Abdominal Distension/Bowel function	3	8.6
Wound infection	7	20
**Total Complications**	**14**	**40**
**Without Complication**	**21**	**60**

### Length of time with stoma or age of patient does not impact on the risk of developing complications following reversal surgery

**Figure 4.**
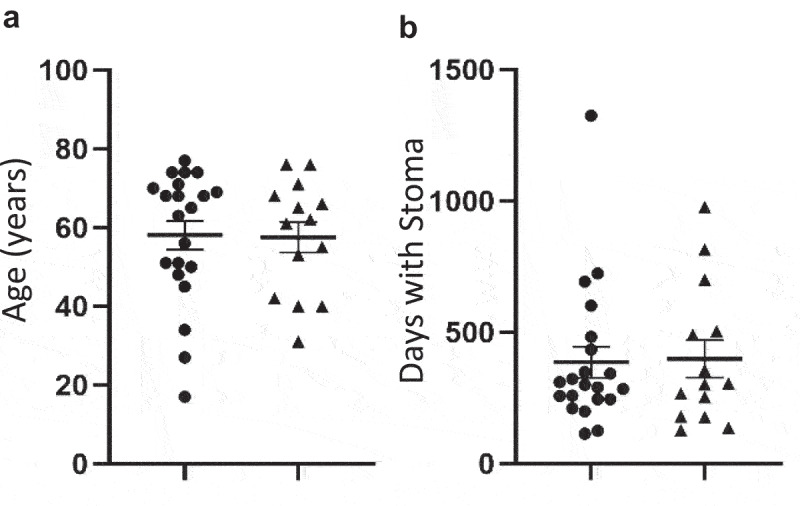
(a) Age and (b) Days with Stoma in *n* = 35 patients without (●) or with (▲) post-operative complications. Lines represent Mean ± SEM.

Patients identified that the most important question to them is whether a delay in stoma reversal surgery impacts upon the risk of post-operative complications. In this study, the average time with a stoma was 392 (±264) days, and the average age of participants was 58 (±16), in line with national averages. We found no increase in the length of time with stoma, or age, in patients that experienced complications (Figure 4a,b). We also observed no correlations between degree of fibrosis or atrophy, as measures of intestinal physiology and potential dysfunction, and the length of time the stoma was in place (Figure 1d,e and f).

### Increased loss of microflora, but not degree of fibrosis and atrophy, impact on the risk of developing complications following reversal surgery

**Figure 5.**
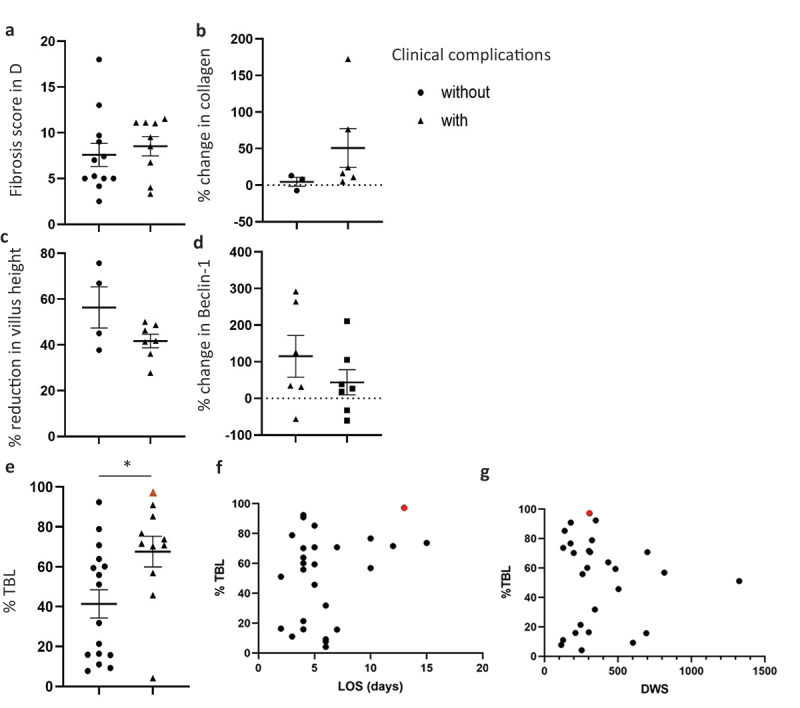
Increased loss of total bacterial load (TBL), but not atrophy or fibrosis, is linked to incidence of post-operative complications. Fibrosis (a) *n* = 12 vs. *n* = 9 n.S., (b) *n* = 3 vs. *n* = 6 n.S.) and atrophy (c) *n* = 4 vs. *n* = 7 n.S., D: *n* = 6 vs. *n* = 7 n.S.) or average % reduction in TBL (E: *n* = 16 vs. *n* = 11, *p = 0.019) in patients without (●) or with (▲) post-operative complications. Mean ± SEM. (f) Length of hospital stay (LOS) vs. %TBL, *n* = 27, *p* = 0.134. (g) Days with stoma (DWS) plotted against % total bacterial load (%TBL, *n* = 27). Red triangle or circle represents patient with anastomotic leak (▲,⌂).

**Figure 6.**
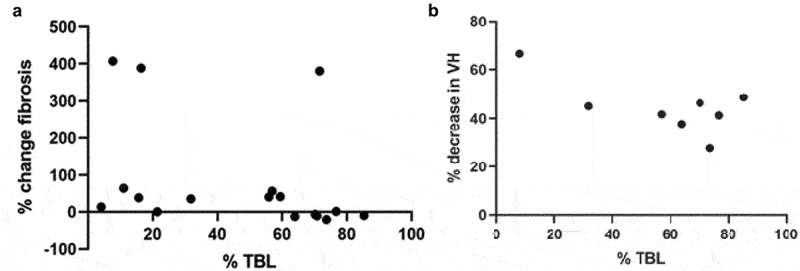
% TBL plotted against % change in a: fibrosis (*n* = 17), b: villus height (VH, *n* = 8). No significant correlations detected.

Using both our measures, we found no increased intestinal fibrosis (Figure 5a,b) or atrophy (Figure 5c,d) in patients experiencing post-operative complications. In our previous publication, we reported a significant loss of total bacterial load (TBL) in defunctioned compared with functional intestine^5^. We now report a significant increase in the loss of TBL (67.6% ±7.7) in patients that experience complications vs. those without (41.4% ±7), Figure 5e. Fortunately, only one patient in our cohort experienced the potentially life-threatening complication of anastomotic leak (with ileus). Interestingly, that individual had the highest % reduction in TBL (97.2%, Figure 5e, red triangle). Lower TBL in the defunctioned bowel also had a tendency to be associated with a longer stay in hospital (Figure 5f), but crucially did not correlate with the length of time the stoma had been in place (5g). Nor did bacterial load influence the levels of fibrosis or atrophy observed (Figure 6a and 6b), leading us to the conclusion that bacteria did not reduce, nor atrophy and fibrosis increase, over time.

### Microbiota diversity

**Figure 7.**
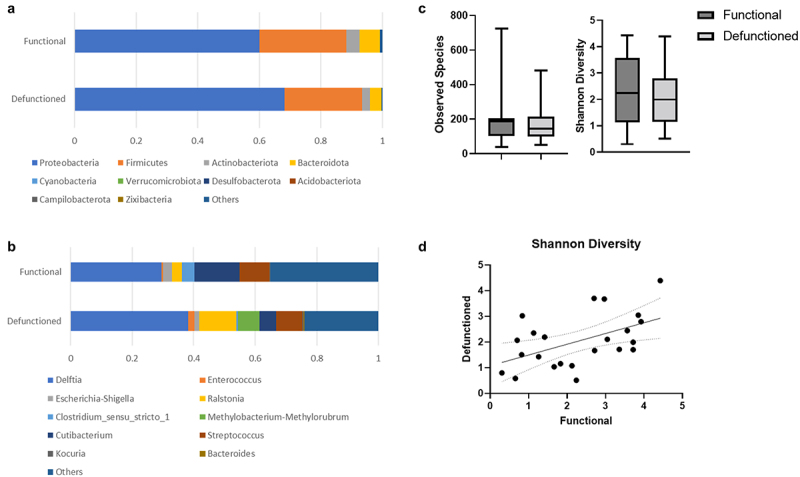
(a) Phylum and (b) Genus abundance in functional and defunctioned ileum in *n* = 23 paired samples. (c) Average Observed species and Shannon diversity score in functional and defunctioned ileum and (d) Shannon diversity in functional vs. Defunctioned ileum (*n* = 23, paired samples, r2 = 0.261, *p* = 0.0127).

In line with our prior qPCR analysis^5^, 16S rDNA sequencing confirmed a reduction in Firmicutes (*p* = 0.05) and an increase in Proteobacteria (*p* = 0.007) phyla in the defuctioned ileum (Figure 7a,b). In contrast with our previous DGGE analysis, although defunctioned ileum may be slightly less diverse (Shannon diversity index average 2.0 vs. 2.3, n.s. Figure 7c), 16S rDNA sequencing revealed 9 out of 23 patients had a more diverse microbiota in the defunctioned than in the functioning portion of their ileum. Interestingly, a correlation was seen (r2 = 0.261, *p* = 0.0127) between the diversity in the functioning and defunctioned ileum within an individual (Figure 7d).

**Figure 8.**
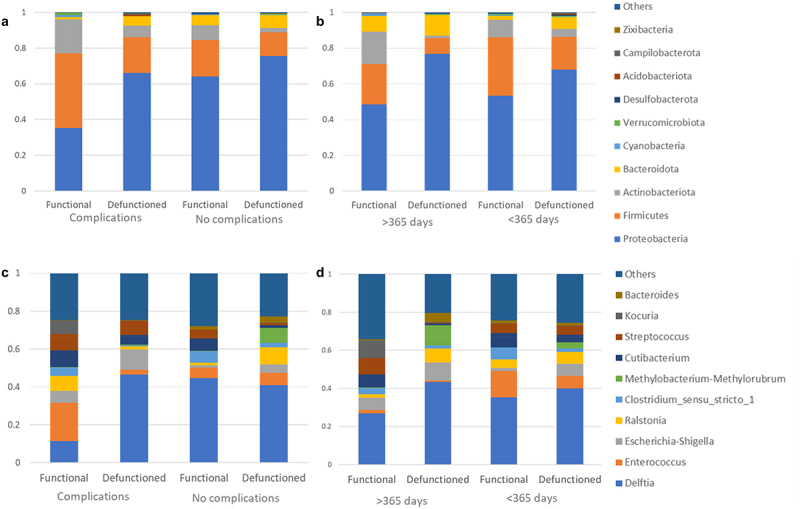
Phylum (a, b) and Genus (c, d) abundance in functional and defunctioned bowel in patients with (*n* = 8) or without (*n* = 15) post-operative complications (a, c) or stoma in place for>365 (*n* = 6) or < 365 (*n* = 17), (b, d).

**Figure 9.**
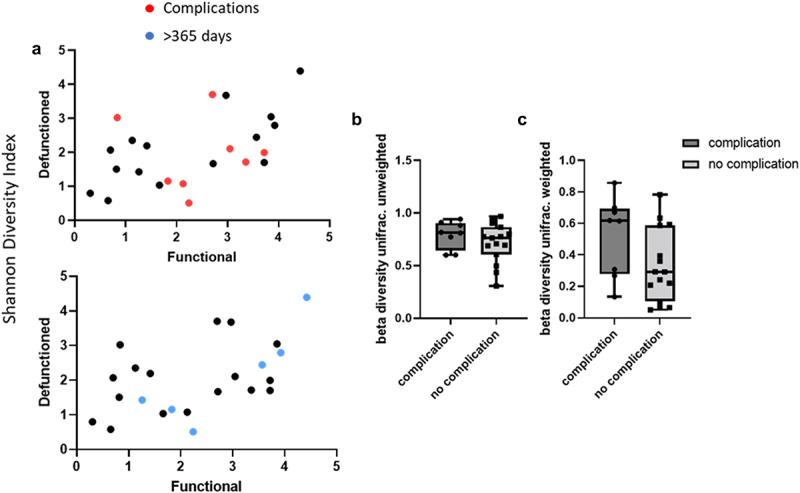
(a) Shannon diversity score in *n* = 23 paired samples.  = complications,  = >365 days since stoma formation. (b) Beta diversity analysis, weighted and unweighted in patients with post-operative complications and without, n.S.

We compared microbiota composition at both the phylum and genus level in the functional and defunctioned ileum of patients who experience complications vs. those who don't (Figure 8a and 8c) and of patients waiting longer than a year for reversal (Figure 8b and 8d). Complication does not have a significant impact on the Shannon diversity index (*p* = 0.4355) (Figure 9). While a higher level of dissimilarity between the functional and defunctioned ileum is seen in those with complications, as reflected by their higher within-subject functional-to-defunctioned ileum beta diversity (unique fraction metric, or unifrac), the difference between with and without complication cohort is not statistically significant (*p* = 0.317 and 0.0749 for unweighted and weighted unifrac).

**Figure 10.**
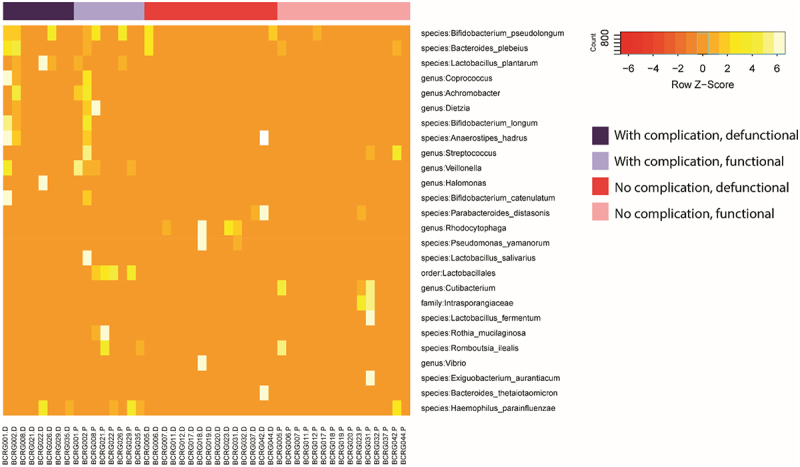
Heat map: pairwise comparisons of Beta diversity.

Initial paired analysis of species abundance revealed 15 species and several genera (Figure 10), that appear significantly different in the ileum of patients that experience complications vs. those that don’t. However, when taking multiple testing into account, only one genus, *Veillonella*, appeared significantly increased in patients that experience complications vs. none.

## Discussion

Our analysis suggests that there is no correlation between the length of time between stoma formation and reversal and measures of pathology such as atrophy or fibrosis. This is supported by the finding that no ongoing Collagen 1 synthesis is occurring at the time of reversal. Not surprisingly, loss of ileal function led to significant upregulation of autophagy, as indicated by the beclin-1 expression. The nutrient-deprived ileum is subject to villous atrophy as well as loss of contractility and smooth muscle strength, which was thought to ultimately contribute to the complications experienced by many patients^4,5^. Autophagy is a crucial process in sustaining cells placed under a range of stressors^15^. Therefore, it was quite surprising that neither of our measures of atrophy (Beclin-1 or villus height) correlated with the length of time a stoma was in place.

Patients are understandably concerned that delaying reversal surgery will increase the risk of developing complications and the possibility that this may lead to a permanent stoma. This concern has become of greater relevance in recent years, due to significant delays in elective surgeries due to the SARS-Cov-2 pandemic. Importantly, we also found no correlation between the risk of developing complications with the length of time a stoma is in place, or patient age, which is somewhat reassuring in the wake of unprecedented delays in reversal surgeries. Fibrosis in the bowel promotes stenosis^9^, and likely contributes to ileus. However, we found no increased incidence of complications, including ileus, in individuals with higher degrees of fibrosis^8^ nor did fibrosis increase with time stoma was in place, or total bacterial load. The only measure we found to strongly correlate with risk of developing complications was a reduced abundance of commensal bacteria, adding to the mounting evidence that the microflora supports the health of the defunctioned bowel. Thankfully, only one case of the potentially fatal complication of anastomotic leak (with ileus) was recorded in our cohort. Interestingly, this was also the patient that was found to have the biggest decrease in TBL, further supporting the protective role of intestinal microflora against anastomotic leak, the most serious complication of this type of surgery^14^.

Although the total number of bacteria is significantly decreased in the defunctioned bowel^5^, 16S rDNA sequencing, in contrast to our previous DGGE analysis^5^, revealed that the diversity of species is not significantly different. Sequencing also revealed a correlation between the diversity of microflora in the functioning and defunctioned ileum within an individual, and that diversity does not correlate with incidence of complications following reversal. Interestingly, several of the species (*Bifidobacterium longum*, *Lactobacillus plantarum*, *Bifidobacteria pseudolongum*, and *Lactobacillus salivarius*) are indicated to be different in patients that experience complications vs. none, are regarded as beneficial probiotic bacteria. However, after when taking into account multiple testing, only one genus *Veillonella* was significantly increased in patients that experience post-surgical complications. Members of this genus are strictly anaerobic, and evidence suggests that some species may play a role in the pathogenesis of inflammatory bowel disease^16^.

A recent study trialed the efficacy and safety of preoperative stimulation of different loops with probiotics prior to ileostomy closure^17^ and whilst safe, they reported limited benefit. Despite the outcome of this small, randomized trial using a commercially available probiotic supplement, the stimulation of the defunctioned intestine should be further investigated. The study administered four strains of *Lactobacillus* and 3 *Bifidobacteria*^17^, designed to be administered orally. These may not be the optimal species to re-stimulate ileal function as the dominant genera in the small intestine are *Veillonella, Enterobacteriaceae, Bacteroides*, and *Prevotella*^18^. Perhaps more of significance; the probiotics were administered in saline, without any sustenance for their establishment and growth^17^. A more effective, and perhaps safer, method may be to administer prebiotics, to stimulate the growth of existing species, rather than attempt to introduce new species into such a specific niche. Our results demonstrating that number, rather than constituents of the microbiota, have a beneficial effect support this statement. Prebiotics are potentially more effective at establishing a more sustainable gut microbiota^19,20^ and may be more effective at reestablishing a microflora that successfully re-stimulates the defunctioned intestine. Future trials should work to optimize ways of reestablishing the microbiota in defunctioned bowel prior to surgery.

## Methods

### Study inclusion criteria

The current study was approved by the North West Research Ethics Committee (13/NW/0695) and conducted in accordance with the Health Research Authority guidelines. A cohort of 44 patients undergoing loop ileostomy reversal surgery at Lancashire Teaching Hospitals NHS Trust (Lancashire, UK) were eligible to participate and consented to the study. Patients who had ongoing bowel pathologies, such as inflammatory bowel disease, or had antibiotic treatments within the last 3 months, were deemed ineligible.

Clinical data was obtained from 35 patients for the total duration of hospital stay. Demographics and clinical data were recorded including age, sex, post-operative blood data, and days since ileostomy formation. The assessed clinical outcomes were length of hospital stay and incidence (and type of) of post-operative complications.

### Histological analysis

Fibrosis scoring: Coded sections were stained with Sirius red to visualize collagen accumulation and scored for fibrosis using a validated scoring system as detailed in^21^. Briefly, the experimental sections were compared with Sirius red stained sections of normal small intestine and scored on a scale of 0–5 for increased extent of collagen deposition throughout different layers of the bowel wall, multiplied by percent of section involved (1=<25%; 2 = <50%, 3 = <75% and 4 = <100%), or intensity of staining (1 = no increase and 2 = increased intensity). In prior studies^21^, fibrosis scores showed highly significant correlation with biochemical measures of fibrosis based on collagen mRNA or protein.

Villus height measurement: Tissue biopsies from functioning and defunctioned intestine were examined using a Motic BA210E microscope at 200× magnification. Scale was calculated using the line drawing function to mark the known distance between two hash marks on a graticule. A minimum of 10 villi from each region were identified and measured. The mean villous height and percentage change was then calculated for each patient.

### qPCR

Collagen Type 1α1 (*COL1A1*) mRNA was quantified using qPCR primers forward :CAGATCACGTCATCGCACAAC, reverse: GAGGGCCAAGACGAAGACATC and normalized to rplp0 forward: GCAATGTTGCCAGTGTCTG and reverse: GCCTTGACCTTTTCAGCAA and Syber Green Master Mix (4309155, Thermo Fisher Scientific). Cycling conditions were 94°C for 2 min, 40 × 94°C for 30 s, 62°C for 1 min and 72°C for 1 min and analysis performed using the ∆∆Ct method.

### Determination of total bacterial load

Total bacterial load was measured via qPCR, using universal eubacterial 16S rRNA primers, Uni334F, and Uni514R (^5^, Table S1) 29. A standard curve constructed for bacterial enumeration using pCR2.1 Topo-TA plasmid vector, containing a cloned portion of the 16S rRNA gene. Bacterial quantification relates to total 16S rRNA gene copy number and not colony forming units or cell counts.

### Western blots

Mini-PROTEAN TGX gels (Bio-Rad #4569036) were used according to the manufacturers instructions. Proteins were transferred onto nitrocellulose membranes and incubated overnight in 2% BSA with various antibodies. Collagen Type 1, 1:1000 (NB600–408, Novus Biologicals), normalized to total protein on a stain-free gel, or Beclin-1 (Cell Signalling #3495) normalized to β-actin (Cell Signaling #3700). Anti-rabbit or mouse secondary antibodies (Cell Signaling #7076 and #7074) and ECL substrate was used to visualize proteins on the ChemiDoc System (Bio-Rad) and densitometric analysis performed using Image Lab software (Bio-rad).

### 16S rDNA sequencing

DNA extracted^5^ from functional and defunctioned ileum of 23 patients was sent for 16S rDNA illumina sequencing (Novogene). Differential abundance analysis data was analyzed using ANCOM-BC2 software package (Bioconductor).

### Scatterplot matrix

A scatterplot matrix was performed to examine linear correlations between multiple variables. Scatterplot matrix analysis was executed utilizing RStudio software (Version 4.1.1, RStudio Inc. Boston, MA), and the function package ‘GGally’ extension to ‘ggplot2’.

### Statistical analyses

Unless otherwise stated, statistical analyses were performed using SPSS Statistics Desktop (Version 28, IBM). *p* ≤ 0.05 was deemed statistically significant (* *p* ≤ 0.05; ** *p* ≤ 0.01; *** *p* ≤ 0.001). Statistical analysis between beta-diversity of complications and no complications cohorts were carried out in R through linear models (lm).

## Data Availability

The data that support the findings of this study are openly available in Lancaster University Pure at https://doi.org/10.17635/lancaster/researchdata/508.
